# Molecular Mechanisms Underlying TDP-43 Pathology in Cellular and Animal Models of ALS and FTLD

**DOI:** 10.3390/ijms22094705

**Published:** 2021-04-29

**Authors:** Alistair Wood, Yuval Gurfinkel, Nicole Polain, Wesley Lamont, Sarah Lyn Rea

**Affiliations:** 1 School of Molecular Science, University of Western Australia, Nedlands 6009, Australia; Alistair.Wood@murdoch.edu.au; 2 Centre for Molecular Medicine and Innovative Therapeutics, Murdoch University, Health Research Building, Discovery Way, Murdoch 6150, Australia; 34219673@student.murdoch.edu.au (Y.G.); Nikki.Polain@murdoch.edu.au (N.P.); 3 Perron Institute for Neurological and Translational Science, Centre for Neuromuscular and Neurological Disorders, University of Western Australia, Nedlands 6009, Australia; Wesley.Lomont@curtin.edu.au; 4 Hub for Immersive Visualisation and eResearch, Curtin University, Bentley 6102, Australia; 5 Harry Perkins Institute of Medical Research, Centre for Medical Research, University of Western Australia, Nedlands 6009, Australia

**Keywords:** amyotrophic lateral sclerosis, frontotemporal lobar degeneration, TARDBP/TDP-43, RNA metabolism, mitochondrial dysfunction, proteostasis, proteinopathy

## Abstract

Amyotrophic lateral sclerosis (ALS) and frontotemporal lobar degeneration (FTLD) are neurodegenerative disorders that exist on a disease spectrum due to pathological, clinical and genetic overlap. In up to 97% of ALS cases and ~50% of FTLD cases, the primary pathological protein observed in affected tissues is TDP-43, which is hyperphosphorylated, ubiquitinated and cleaved. The TDP-43 is observed in aggregates that are abnormally located in the cytoplasm. The pathogenicity of TDP-43 cytoplasmic aggregates may be linked with both a loss of nuclear function and a gain of toxic functions. The cellular processes involved in ALS and FTLD disease pathogenesis include changes to RNA splicing, abnormal stress granules, mitochondrial dysfunction, impairments to axonal transport and autophagy, abnormal neuromuscular junctions, endoplasmic reticulum stress and the subsequent induction of the unfolded protein response. Here, we review and discuss the evidence for alterations to these processes that have been reported in cellular and animal models of TDP-43 proteinopathy.

## 1. Introduction

Amyotrophic lateral sclerosis, the most common form of motor neuron disease, is characterized by the progressive loss of upper and lower motor neurons, leading to death 2–4 years post-diagnosis, usually due to respiratory failure [1]. Frontotemporal lobar degeneration is caused by the progressive loss of neurons in the frontal and temporal lobes of the brain and is the second leading cause of early-onset dementia worldwide [2]. ALS and FTLD exist on a disease continuum as they share clinical, neuropathological, and genetic similarities [2]. Of note, 12.5% of FTLD cases have concomitant motor neuron disease, with a further 30% showing signs of motor neuron dysfunction [3]. Similarly, 50% of ALS cases have signs of cognitive decline, with 20% fitting the diagnostic criteria for FTLD [4]. The pathobiology of ALS can include neuroinflammation, gliosis, glutamate-mediated excitotoxicity and subsequent increased intracellular calcium, endoplasmic reticulum (ER) stress, impairments to protein homeostasis (proteostasis) and axonal transport, mitochondrial dysfunction, free radical accumulation due to increased oxidative stress and increased apoptosis [5,6]. The underlying causes of these disease processes are largely unknown but may, at least in part, be linked with the buildup of aggregated proteins, such as TDP-43, superoxide dismutase 1 or C9orf72 dipeptide repeats caused by hexanucleotide G_4_C_2_ repeat expansion [7].

A key characteristic of neurodegenerative diseases is the identification of abnormal protein aggregates in neurons [8]. In up to 97% of ALS cases and the majority of non-tau-related FTLD cases, these aggregates contain the human nuclear ribonuclear protein TAR DNA-binding protein 43 (TDP-43), suggesting similar molecular mechanisms for the two diseases [9,10,11]. Thus, ALS- and TDP-43-associated FTLD (FTD-TDP) are considered TDP-43 proteinopathies. Mutations in *TARDBP,* coding for mutant TDP-43 proteins, are a rare cause of ALS and FTLD [12]. Four percent of fALS and 1.5% of sALS are caused by mutations in *TARDBP,* with most being missense mutations that affect the C-terminal domain [13]. Mutations have very rarely been identified in FTLD cases, with and without co-morbid ALS [14,15]. Of note, the TDP-43 observed in degenerating neurons in ALS and FTLD tissues in both mutation and non-mutation cases is abnormally cytoplasmic, hyperphosphorylated, ubiquitinated and often cleaved, giving rise to C-terminal fragments (CTFs) [16,17,18]. TDP-43 is primarily a nuclear protein, thus the cytoplasmic mislocalization and aggregation of TDP-43 are considered important pathogenic mechanisms in ALS and FTLD. However, neuronal TDP-43-positive intranuclear inclusions are also observed [19]. The aggregation of TDP-43 may be a primary disease process, or a byproduct of a distinct initiating pathological process. However, the removal of TDP-43 from its functional site in the nucleus is likely to impact diverse biological processes that could include disruptions to mRNA splicing, cryptic exon retention, polyadenylation, nuclear export, localization, and translation [11]. TDP-43 has also been implicated in axon outgrowth [20], axonal transport [21], mitochondrial function [22], and autophagy [23]. Thus, while the exact contribution of TDP-43 cytoplasmic mislocalization and aggregation to disease progression is unclear, both loss and gain of function are thought to be involved. In this review, we will summarize and discuss the molecular mechanisms that have been identified in cellular and animal models of TDP-43 proteinopathies.

## 2. Pathogenic Mechanisms of TDP-43 in Cell and Animal Models

While TDP-43 pathology is identified in almost all cases of ALS and non-tau-related FTLD, the mechanisms underlying pathogenicity are varied and incompletely understood. 

### 2.1. Neuron Morphology and Axonal Degeneration

Both conditional knockout [24] and TDP-43 overexpression [25,26,27] cause neuronal degeneration and reduced cell viability. Overexpression of TDP-43 may contribute to ALS pathogenesis via the formation of insoluble aggregates, leading to impaired axonal transport of essential energy metabolizing organelles such as the mitochondria. This may result in cytotoxicity and neuronal apoptosis. Knockout of TDP-43 may result in transcriptional irregularities or aberrant mRNA splicing (Kitamura, A., 2018). The presence of various mutations increases the stability of TDP-43, leading to higher expression levels, which may contribute to pathogenesis [28]. It is possible that in non-mutation cases, there is a trigger that leads to increased TDP-43 levels that impairs neuronal health. Alternatively, there may be a trigger for TDP-43 translocation and aggregation in the cytoplasm. TDP-43 mutant proteins (p.M337V and p.Q331K) overexpressed in rat cortical neurons mislocalize into cytoplasmic aggregates. These neurons, and also those overexpressing wild-type TDP-43, displayed shorter axons and smaller growth cone sizes compared with control cells, suggesting a role for TDP-43 in cytoskeletal regulation [25]. Similarly, overexpression of mutant TDP-43 (p.A315T, p.Q331K or p.M337V), or TDP-43 lacking a nuclear localization signal (ΔNLS), led to abnormal neurite morphology and decreased cortical neuron viability [26]. These studies suggest that the translocation of TDP-43 out of the nucleus is critical to pathogenesis in TDP-43 proteinopathy involving dysfunctional axonal growth, decreased axonal innervation and neuronal viability. 

Axon outgrowth is essential for neuronal communication and innervation of action potentials in neuromuscular junctions (NMJs). TDP-43 has been implicated in axon outgrowth and the denervation of NMJs, causing defective synaptic transmission, and impacting cell survivability and conductivity. The overexpression of wild-type or mutant TDP-43 (p.M337V or p.Q382T) in mouse primary neurons caused impaired axon outgrowth. This was shown to be dependent on the C-terminal domain [29]. Further, there was an increased number of degenerated axons in the ventral root of mice expressing TDP-43^A315T^ [30]. However, overexpression of TDP-43^A315T^ in the primary cortical neurons of a distinct transgenic mouse did not significantly affect dendrite or axon outgrowth [31]. Axonal destruction is a prominent feature of neurodegeneration and can also be caused by injury via a process termed Wallerian degeneration, where an injury at a particular site affects all axons simultaneously [32]. A critical gene in this pathway is *sterile alpha and TIR motif-containing 1*. Deletion of this gene in a TDP-43^Q331K^ mouse model of ALS-FTLD reduced the degeneration of motor axons and the denervation of NMJs, thus implicating Wallerian-like pathways in the axonal degeneration observed in ALS [33]. Denervation of NMJs is a characteristic feature of ALS. In TDP-43^Q331K^ transgenic mice, defective synaptic transmission at the NMJ is observed at age 3 months, preceding the motor dysfunction that was observed at age 10 months [34]. This fits with the theory that ALS is a ‘dying back’ disease, where denervation and degeneration occur before motor neuron loss [35]. Further implicating TDP-43 in NMJ function, innervation of NMJs in the soleus muscle progressively decreased with age in a knockin TDP-43^N390D^ mouse [36]. Expression of TDP-43^A315T^ in mouse cortical neurons decreased the localization of the α-amino-3-hydroxy-5-methyl-4-isoxazolepropinionic acid (AMPA) glutamate receptor subunit GluR1 at dendritic spines, which was associated with a reduction in the presynaptic marker synaptophysin [31]. Thus, denervation of NMJs and dysregulated synaptic function may be related to declining neuronal function in ALS and FTLD. TDP-43 mislocalization may play a critical role in causing ALS and FTLD axonal dysfunction.

### 2.2. Post-Translational Modifications

TDP-43 is primarily a nuclear protein [11]. Its translocation to the cytoplasm is associated with various post-translational modifications (PTMs) including cleavage and aggregation, acetylation, ubiquitination, SUMOylation and phosphorylation, which may lead to toxic gain of function in the cytoplasm. 

#### 2.2.1. Cleavage

**Figure 1:**
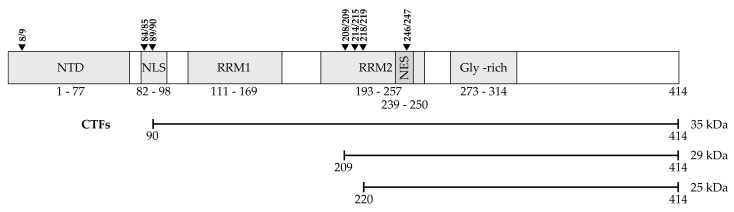
TDP-43 domain structure, potential caspase cleavage sites and CTFs. TDP-43 domain structure consists of an N-terminal domain (NTD), a nuclear localizing sequence (NLS), two RNA recognition motifs (RRM1 and RRM2), a glycine-rich domain and a nuclear export sequence (NES). Caspase-mediated cleavage sites are indicated with black triangles and can occur between amino acid residues 8/9, 84/85, 89/90, 208/209, 214/215, 218/219 and 246/247. The major CTFs that have been identified in patient tissues are indicated [41,42,43].

While TDP-43 is autoregulated at a transcriptional level, it is also degraded by both the ubiquitin-proteasome system (UPS) and the autophagy-lysosome pathway, hereafter autophagy [37]. It has been suggested that cleavage, or fragmentation, may be a prerequisite to proteolytic degradation [38]. TDP-43 can be cleaved by caspase-3 and calpains [39]. There are multiple potential cleavage sites (Figure 1) that give rise to 15, 25 and 35 kDa CTFs [40,41,42,43]. The consistent identification of phosphorylated and fragmented TDP-43 in affected tissues suggests that cleavage and phosphorylation of accumulated TDP-43 may be part of the pathological process in ALS and FTD-TDP [16,17,44,45]. Of note, while CTFs are observed in the insoluble fractions of brain tissue from ALS and FTLD patients, they are either not detected or are only faintly detected in spinal cord tissue [18,44]. 

##### C-Terminal Fragments

When expressed in HEK293 cells, a 35 and a 25 kDa CTF formed cytoplasmic aggregates in 25% and 75% of cells, respectively [45]. Expression of a CTF consisting of aa162-414 was phosphorylated and formed aggregates, and while cytotoxicity was observed after 7 days of expression, it was less cytotoxic than overexpression of full-length TDP-43, which was not phosphorylated and did not aggregate [27]. Overexpression of TDP-43-ΔNLS did not aggregate or cause significant cell death. Thus, cleavage appears to precede aberrant TDP-43 phosphorylation and cytoplasmic aggregation but may not be required for toxicity. Expression of a 25 kDa CTF (aa208-414) in a transgenic mouse model induced hyperphosphorylation, insolubility and a punctate accumulation of TDP-43 in the cytoplasm, without inducing the formation of large inclusion bodies. This was accompanied by neuron loss in the hippocampus and astrogliosis [46]. However, earlier studies showed that while a 25 kDa CTF was more highly phosphorylated than full-length TDP-43, the formation of inclusions and cytotoxicity was not dependent on CTF phosphorylation [45]. Furukawa et al. (2011) showed that the formation and localization of inclusion bodies, or aggregates, depends on the cleavage site (Table 1) [47]. Single nuclear inclusions were observed for some CTFs (aa188-414, aa193-414, aa198-414 and aa203-414) whilst others (aa208-414, aa213-414, aa220-414 and aa225-414) formed multiple cytoplasmic inclusions [47]. These CTFs all had increased insolubility compared to full-length TDP-43 and were phosphorylated. Proteins that corresponded to cleavage in more N-terminal regions (aa167-414, aa173-414, aa178-414, aa183-414) had similar insolubility when compared to full-length TDP-43 and rarely formed inclusion bodies. The authors showed that phosphorylation was not required for insolubility, as TDP-43 aa208-414 with Serine to Alanine substitutions was insoluble. Thus, phosphorylation may be a post-aggregation phenomenon [47]. Brady et al. (2011) also showed that aggregation of TDP-43 with Serine409/410 to Alanine substitutions, while reduced by ~15%, still occurred [48]. Taken together, it appears that it is cytoplasmic aggregation, rather than the characteristic hyperphosphorylation of TDP-43, that is critical to cytotoxicity. Wei et al. (2017) found that there may be two mechanisms responsible for the increased propensity to aggregate and the toxicity of CTFs: abolishment of the RRM2 structure, or release of the C-terminal domain [49].

Translocation of TDP-43 out of the nucleus causes loss of function (see Section 2.2.1), while the formation of cytoplasmic aggregates containing CTFs are thought to give rise to toxic gain of function. When overexpressed, the TDP-43^A315T^ mutant protein is fragmented into persistent CTFs that resist proteolytic degradation [50]. The p.Q343R [51], p.G348C, p.R361S and p.N390D [52], p.M337V, p.N345K and p.I383V [53], p.A382T and p.S393L [54] mutant proteins also generate cleavage products of 25, ~28 or 35 kDa (Table 1). Overexpression of a 25 kDa CTF consisting of amino acids (aa) 208-414 is toxic to primary cortical neurons, M17 neuroblastoma and HEK293 cells [45,55,56]. A further study showed that a 25 kDa CTF was more susceptible to aggregation than a 35 kDa CTF when overexpressed. The 25 kDa CTF was also significantly more cytotoxic than full-length TDP-43, whereas the 35 kDa CTF was not [56]. However, overexpression of CTFs did not decrease cell viability compared to those expressing full-length TDP-43 in either NSC-34s or SH-SY5Ys, yet they did impair neurite outgrowth [27,57]. The toxicity of CTFs generated by caspase cleavage is dependent on the cytoplasmic translocation of TDP-43, as the fusion of an NLS to a 25 kDa CTF attenuated Neura2A cell death [56]. The importance of the NLS to TDP-43 proteinopathy and toxicity has been further demonstrated in ΔNLS mouse models [58,59,60]. A mouse model with inducible hTDP-43ΔNLS expression showed cytoplasmic accumulation of insoluble, phosphorylated TDP-43. The mice exhibited significant neurodegeneration with neuron loss, muscle denervation and progressive muscle impairment. Importantly the induced return of nuclear TDP-43 expression rescued the neurodegeneration phenotype [60]. By contrast, a distinct hTDP-43ΔNLS mouse model only rarely showed phosphorylated cytoplasmic aggregates yet also gave rise to a phenotype resembling ALS. Pathogenesis appeared to be due to reduced nuclear TDP-43 function, as profound gene expression changes were observed [58]. Sasaguri et al. (2016) showed that mutation of a single residue in the NLS, but not deletion of the extreme N-terminus (aa 1–9), caused cytoplasmic accumulation of TDP-43 [59]. Using sequentially deleted N-terminal proteins, Nonaka et al. (2009) found that aa 1–161 within the RNA recognition motif 1 (RRM1) domain are required for the regulation of exon skipping in the *cystic fibrosis transmembrane conductance regulator* gene [42]. Thus, CTFs generated due to mutations, that do not contain these N-terminal amino acid residues may lose the ability to regulate RNA as well as gaining toxic cytoplasmic functions.

#### 2.2.2. Acetylation

Acetylation is an enzyme-mediated process whereby an acetyl group is moved from one molecule to another to alter intracellular localization, protein–protein interactions or tag proteins for degradation. Various TDP-43 RNA regulatory functions are impaired due to acetylation. Specifically, RNA binding is inhibited by post-translational acetylation at lysine residue 145 (Lys-145), which sits within the RNA-binding domain [61]. This led to a reduction in splicing function. Acetylation of TDP-43 induced the aggregation of insoluble, hyperphosphorylated TDP-43 within neuronal cells. TDP-43 became insoluble and hyper-phosphorylated after cytoplasmic acetylation was induced through the use of single and double acetylation mimics within TDP-43-∆NLS [61]. Insolubility and hyper-phosphorylation are characteristic mechanisms of pathological TDP-43 inclusions displayed in ALS and other TDP-43 proteinopathies. Cohen et al. (2015) analyzed spinal cord tissue from ALS patients and found acetylated (Lys-145) and phosphorylated (p-Serine 409/410) TDP-43 lesions. Brain tissue from FTD-TDP patients failed to show the same result, with only phosphorylated TDP-43 inclusions present. The absence of acetylation may be explained by the fact that TDP-43 aggregates in FTD typically consist of CTFs beginning at residue Arg-208, as such the residue that is acetylated (Lys-145) is not present [61]. A further study using ALS brain tissue showed no acetylation at Lys-145 or Lys-192, instead reporting acetylation at Lys-82 [62]. This indicates that there may be heterogeneity between patients.

Sanna et al. (2020) show that TDP-43, via its RRM1 and RRM2 domains, interacts with histone deacetylase 6 (HDAC6). The RRM domains contain the two major acetylation sites in TDP-43 Lys-142 and Lys-192 [63]. The authors used cell and drosophila models to show that treatment with pan-HDAC inhibitors was protective against the toxicity induced by overexpression of wild type or mutant TDP-43. Therefore, acetylation of TDP-43 may be a potential therapeutic target in ALS and FTLD [63]. A new study highlighted the mechanisms underlying TDP-43 acetylation related pathology. Yu et al. (2021) showed that TDP-43 that was either unable to bind to RNA, or TDP-43 that was acetylated within the RRMs underwent phase separation into anisosomes [64]. TDP-43 acetylation promoted the formation of these liquid spherical shells with liquid cores. Of note, when ATP levels were lowered, as can be seen in post-mortem tissues, these anisosomes transformed into protein aggregates, suggesting that anisosomes may be antecedents of the pathological aggregates identified in patient tissues [64].

#### 2.2.3. Ubiquitination

Attachment of ubiquitin to target proteins is a well-known signal for protein degradation. TDP-43 ubiquitination was one of the first signature modifications to be associated with ALS and FTLD [18,42]. A mutagenesis study utilized mass spectrometry to show that Lys-84, Lys-95, Lys-160, Lys-181 and Lys-263 are TDP-43 ubiquitination sites, with Lys-84 implicated in the regulation of TDP-43 nuclear import. Lys-84 and Lys-95 were investigated further and shown to have no effect on TDP-43 solubility [65]. The authors also investigated 15 TDP-43 mutant proteins and showed that only one, p.K263E, was heavily modified with ubiquitin. The remaining mutant proteins were ubiquitinated to a similar extent as the wild-type protein. The pathological 35 kDa CTF (aa193-414) contains 4 Lysine residues. The authors showed that this fragment is heavily ubiquitinated and that removal of at least 3 lysines was required to suppress ubiquitination [65]. However, Lys-192 substitution with an Arginine decreased ubiquitination. Of note, in the context of full-length TDP-43, ubiquitination of the C-terminal domain was not observed. This may be explained if ubiquitination occurs following cleavage. Of note, p.Q331K and p.N345K mutations did no display increased ubiquitination [65].

#### 2.2.4. SUMOylation

The small ubiquitin-like modifier (SUMO) pathway involves enzymatic PTM, resulting in the covalent attachment of SUMO proteins to a target protein’s lysine residues [66]. SUMOylation is a reversible and dynamic pathway that can compete with ubiquitin to enact gene transcription modifications and alter subcellular localization or protein turnover. Importantly SUMOylation plays a role in the cells response to oxidative stress, hypoxia and glutamate excitotoxicity as well as proteasome impediment, all of which are aberrant processes linked to motor neuron degeneration in ALS [66]. Various studies have shown that SUMOylation of fused in liposarcoma, superoxide dismutase and TDP-43, al implicated in ALS pathogenesis, occurs [67]. Overexpressed full-length TDP-43 or a splicing variant resulted in an increase in the level of SUMO-2/3 and ubiquitin detected in cells, suggesting that SUMOylation and ubiquitination may be interlinked. SUMO-2/3 was predominantly colocalized with TDP-43 primarily in nuclear inclusions [68]. Maurel et al. (2020) identified a unique consensus site for SUMOylation at lysine-136 of TDP-43 [69]. They further show that replacement of this residue with an arginine altered the intracellular localization of TDP-43 aggregates from the cytoplasm to the nucleus, indicating that SUMOylation of this residue is critical to TDP-43 cytoplasmic localization [69]. However, it is not known whether SUMOylation of TDP-43 creates a toxic species.

#### 2.2.5. Phosphorylation and Aggregation

In addition to PTMs such as ubiquitination, oxidation and acetylation, phosphorylation, the generation of CTFs, cytoplasmic accumulation and mutations are all factors that affect TDP-43 aggregation [70]. Various CTFs (17 to 26 kDa) mislocalize to the cytoplasm [16,47] and are associated with TDP-43 phosphorylation and sarkosyl insolubility [16].

##### Mutant TDP-43 Proteins

Expression of mutant TDP-43 (p.D169G, p.K181E, p.K263E [71] and p.Q331K [55]) leads to co-aggregation with endogenous wild-type TDP-43, whereas overexpression of wild-type TDP-43 exacerbates p.Q331K aggregation [72]. In primary neurons, overexpressed TDP-43^A315T^ was mainly present in the insoluble fraction, whilst overexpressed wild-type TDP-43 was primarily soluble [50]. Some ALS-associated TDP-43 mutant proteins, p.A315T and Y374X, but not others, p.M337V or p.A382T, were associated with increased insolubility (Table 1). Yet, when introduced into a TDP-43 CTF (aa235-414), p.M337V and p.Y374X increased the insolubility of the CTF, whereas p.A315T and p.A382T did not [47]. Another study investigated the effects of 14 ALS mutations on CTF (aa162-414) inclusion formation. All increased the number of aggregates observed. However, only half (p.D169G, p.G294A, p.Q331K, p.M337V, p.Q343R, p.N390D and p.N390S) reached significance when compared with the wild-type CTF [42]. However, Fallini et al. (2012) reported that while p.M337V and p.A382T TDP-43 mislocalize to the cytoplasm, they did not exhibit increased aggregation [29]. Watanabe et al. (2013) tested the solubility and localization of 18 ALS mutant TDP-43 proteins and found that while all had decreased solubility, and 11 were less nuclear compared with wild-type (Table 1), neither measure correlated with either disease onset or duration. However, increased stability (half-life) of mutant proteins was associated with earlier disease onset [28].

##### Oligomerization

The formation of TDP-43 into oligomers is an intermediary step that occurs before aggregate formation [76]. Oligomerization of TDP-43 transpires via its N-terminus and enables it to function as a modulator of RNA splicing [77]. Full-length TDP-43 forms oligomers in FTD-TDP patients that are toxic [78]. However, dysfunctional oligomerization of mutant TDP-43 can lead to the formation of insoluble aggregates and result in cytotoxicity. Abnormal disulphide-crosslinked TDP-43^A315T^ oligomers were isolated from the cortical tissue of mice expressing this protein under the prion promoter, and high-molecular-weight poly-ubiquitinated TDP-43 aggregates were isolated from the spinal cord [30]. When expressed in motor neuron-like cells, both wild-type and mutant (p.Q331K and p.M337V) TDP-43 translocated into cytoplasmic aggregates in response to oxidative stress [79]. The authors found that both cysteine-dependent insoluble oligomers and cysteine-independent aggregates formed. This was not dependent on the presence of a mutation, but rather was reliant on the presence of the N-terminal domain. The authors were able to distinguish between oligomer formation and aggregation. It was shown that 35 and 25 kDa CTFs localize into oligomers when full-length TDP-43 is co-expressed but are observed in large aggregates when an N-terminal deletion protein was co-expressed [79]. Conversely the N-terminal domain is stabilized by disulphide bridges at cysteine residues 39 and 50, and dimerization prevents cytoplasmic aggregate formation [80]. A TDP-43^C39S/C50S^ double mutation almost completely abolished oligomer formation. Thus, oxidation of these residues is important for the initiation of oligomerization. Of note, the substitution of any of the other 4 cysteine residues in TDP-43 to serine or alanine caused hyper-aggregation [79]. Aggregation of mutant proteins, such as TDP-43, is viewed as toxic proteinopathy in neurodegenerative diseases such as ALS. It is currently controversial as to whether aggregates are inherently toxic or are byproducts that act as toxicity markers.

##### Aggregate Toxicity

There has been debate as to whether TDP-43 aggregation is by itself toxic. Phosphorylation of TDP-43 by casein kinase 1ε (CK1ε) appears to increase the propensity of TDP-43 to aggregate in response to oxidative stress [81]. Indeed, expression of a hyperactive CK1ε increased TDP-43 aggregation and this was linked with decreased cell viability [82]. However, Barmada et al. (2010) showed that it is TDP-43 cytoplasmic mislocalization, rather than aggregation, that is the predictor of neuronal death [83]. Consistent with this finding, Sasaguri et al. (2016) observed that TDP-43-ΔNLS rarely aggregated, however its overexpression was toxic to primary neurons [59]. However, as discussed previously, expression of a ΔNLS construct in SH-SY5Y cells did not aggregate and was not cytotoxic [27]. Of note, a mouse model overexpressing TDP-43-ΔNLS displayed a neurodegeneration phenotype, yet TDP-43-ΔNLS was only observed in cytoplasmic aggregates in a small percentage of cells [60]. Overexpression of TDP-43-ΔNLS caused cytoplasmic co-aggregation/homodimerization of the deletion protein with the endogenous full-length protein. This was associated with impaired neurite outgrowth and was dependent on the presence of the extreme N-terminus (amino acids 1–10) [84]. Mutation of the NLS in mice leads to gliosis and motor deficits, whereas deletion of the first 9 amino acids of the N-terminus reduces aggregate formation and toxicity [59]. Thus, the aggregation and toxicity of overexpressed TDP-43 appear to be partially mediated by the extreme N-terminus. However, it may be translocation out of the nucleus that causes toxicity.

Given that the majority of ALS and FTLD cases with TDP-43 proteinopathy are not caused by *TARDBP* mutations, it is important to understand the role of aggregated and mislocalized wild-type TDP-43 in disease pathogenesis. Transfection of SH-SY5Y cells with purified bacterially expressed wild-type TDP-43 aggregates caused decreased cell viability due to an increase in reactive oxygen species (ROS) and exacerbated caspase-3 activation [85]. Overexpression of full-length TDP-43, but not TDP-43-ΔNLS or CTF aa162-414, caused cleavage of poly-(ADP-ribose)polymerase-1 (PARP-1), a well-known substrate of caspase-3 in apoptosis initiation [27]. Further arguing against a role for aggregation of TDP-43 in neurotoxicity, some expression models of wild-type TDP-43 are cytotoxic whilst rarely showing aggregation [25,27]. There have been inconsistent results in mice overexpressing TDP-43. Some mouse models expressing human wild-type TDP-43 cause neurodegenerative phenotypes [86,87], while others did not [72]. A mouse expressing the human TDP-43^A315T^ transgene developed progressive and fatal neurodegeneration, with selective vulnerability of spinal cord neurons and neurons from the frontal cortex. However, TDP-43 aggregates were not present, reinforcing that altered TDP-43 function is required for neurodegeneration to occur, independent of aggregation [74]. 

### 2.3. Loss of Nuclear Function

TDP-43 is primarily located in the nucleus of cells. However, it can also be present in the cytoplasm. This nucleocytoplasmic shuffling is mediated by nuclear localization and nuclear export signals. The loss of nuclear TDP-43 due to abnormal cytoplasmic mislocalization is thought to lead to loss-of-function effects and contribute to disease pathogenicity in ALS [88].

#### 2.3.1. RNA Splicing and RNA Instability

##### Autoregulation

TDP-43 has key roles in the metabolism, processing, alternative splicing and transport of RNA [89]. TDP-43 binds to long clusters of UG-rich sequences, which are found in one-third of transcribed genes. Thus, TDP-43 regulates the processing of up to thousands of transcripts, including that of its own transcript [90]. Autoregulation of TDP-43 establishes a tightly regulated feedback loop. It has been demonstrated that a 2-fold increase or decrease in TDP-43 concentration is sufficient to cause neurodegeneration [90]. TDP-43 recognizes the 3′ untranslated region (UTR) that triggers alternative splicing of the transcript of introns 6 and 7 within the final exon of TDP-43 mRNA, thereby destabilizing the mRNA and ultimately reducing protein expression [90,91]. The splicing event establishes exon–exon junctions which induce the introduction of exon-junction complexes, comprised of eIF4α-III, Magoh, Y14 and UPF3. These complexes stall translation and permit association between the SURF complex (SMG1, UPF1 and eRF1 and 2) and the halted ribosome. The established complex interacts with a stop codon >50 nucleotides downstream, phosphorylating UPF1 and activating nonsense-mediated decay of the transcript [90]. Induced TDP-43 expression decreases the level of endogenous protein expressed. Thus, TDP-43 autoregulates via a negative feedback loop. In mouse models that heterozygously express either human TDP-43 p.A382T or p.G348C, silencing of the mutant allele led to a 1.5-fold increase in expression of the endogenous wild-type protein. Thus, mutant TDP-43 participates in the autoregulation feedback loop [92]. 

##### mRNP Granules

mRNAs are trafficked in dendrites and axons as ribonucleoprotein (mRNP) granules that consist of mRNAs, RNA-binding proteins (RBPs), ribosomes and translation factors [93,94]. mRNP granules are transported by microtubules to deliver mRNA to distal compartments in neurons. TDP-43 accumulates within these granules in the cytoplasm and facilitates RNA trafficking [21]. Expression of TDP-43 mutant proteins (p.M337V and p.A315T) impaired the transport of target mRNAs in mouse cortical neurons, as granules were more often immotile and had an increase in retrograde movement compared with cells expressing wild-type TDP-43 [21]. These findings were confirmed in motor neurons derived from ALS patient-induced pluripotent stem cells (iPSCs) harboring mutations (p.M337V, p.A315T and p.G298S) [21]. In another study, mRNP granules containing ALS-linked mutant TDP-43 (p.G298S and p.M337V) had decreased motility and increased viscosity. The transport of mutant TDP-43 RNP granules was interrupted more than wild-type granules, with stalling or halting observed when granules interacted [95]. The transport and translation of mRNA in neurons are interlinked, with mRNAs remaining inactive until they are unmasked by activation signals. TDP-43 was shown to co-operate with the RNA-binding proteins fragile X mental retardation protein or Staufen1 to regulate anterograde or retrograde transport of mRNP granules, respectively [96]. RNAi-mediated depletion of TDP-43, FMRP or Staufen1 significantly altered the movement of endogenous *Rac1* [96]. Chu et al. (2019) show that TDP-43 regulates the dynamics and kinetics of coupled mRNA transport (microtubule-dependent) and translation in the dendrites of mouse hippocampal neurons [96]. When the RRM domains are unable to bind to RNA toxicity is reduced, indicating that TDP-43 binding to RNA may be an important factor in ALS and FTLD pathogenesis [97,98,99,100].

##### Splicing

TDP-43 has a critical role in repressing splicing to protect the transcriptome [101]. Using a global proteomic approach, TDP-43 was shown to have an extensive network of interaction partners that are known to regulate RNA metabolism, including a nuclear splicing cluster and a translation cluster [102]. TDP-43 associates with factors important for the splicing and transport of RNA [102,103,104]. These interactions can be RNA-binding dependent or independent [102]. Depletion of TDP-43 in mice led to alterations to the level of 601 mRNAs including *fused in sarcoma/translocated in liposarcoma (FUS/TLS), progranulin* and other genes associated with neurodegeneration. Using splicing-sensitive microarrays, TDP-43 was found to be important for the maintenance and splicing of more than 1000 mRNAs. The RNAs that were most reduced by TDP-43 knockdown encoded proteins that are important for synaptic activity [105]. While it seems plausible that a loss of function due to cytoplasmic mislocalization may contribute to ALS and FTLD pathogenesis via reduced RNA regulation, the presence of the p.A315T or p.M337V mutations did not significantly affect the interactome [102].

In mutant mice expressing high levels of TDP-43^Q331K^, which develop adult-onset ALS symptoms, exon exclusion in some pre-mRNAs was enhanced compared to mice expressing human wild-type TDP-43. Of note, TDP-43^Q331K^ increased the normal splicing of some TDP-43 targets, while other targets exhibited a loss of splicing function that mimicked changes observed due to reduced TDP-43 activity [106]. Some exons were misregulated by increased expression of wild-type or mutant TDP-43, but not by TDP-43 deficiency [106]. ALS-associated *TARDBP* mutations near the RRM domains (p.K181E, p.D169G and p.K263E) disrupt RNA binding and also cause sequestration of wild-type TDP-43 in cytoplasmic inclusions, suggesting that RNA binding may be linked to TDP-43 solubility [71]. Of note, inhibition of TDP-43/RNA interactions, mediated by the RRM domains of TDP-43, by a small molecule (rTRD01) partially rescued the locomotor dysfunction observed in a *TARDBP* mutation (p.A315T) drosophila model of ALS [107]. Using two strains of mice generated through *N*-ethyl-*N*-nitrosourea (ENU) mutagenesis, Fratta et al. (2018) were able to identify a mutation-associated (p.F201I) novel splicing event that involves the skipping or inclusion of constitutive exons, referred to as “skiptic” exons, to alter gene expression [108]. The mutations were present within the RRM2 domain (p.F210I) and the C-terminal domain (p.M323K). The p.M323K mutation was shown to affect TDP-43 phase separation, similarly to the ALS-associated p.Q331K mutation. The ENU-induced mutations were shown to have loss (p.F210I) or gain-of-function (p.M323K) splicing effects on exon inclusion in the *CTFR* and *Sortilin-1* genes, while 47 skiptic exons were differentially regulated in the p.F210I strain. The skiptic exons identified in the p.M323K strain were absent in the p.F210I strain. Of note, the p.F210I mutation had similar effects transcriptome wide as shRNA-mediated TDP-43 knockdown. The p.M323K mutation had opposing effects and led to the skipping of constitutive exons in a distinct set of 44 genes, including 7 genes from the UPS [108]. Importantly, in p.M323K mice, a progressive neuromuscular phenotype involving loss of grip strength and muscle force and a significant reduction in motor units was observed. The mice also showed p62 and ubiquitin-positive inclusions, reminiscent of neurodegenerative disease states, and upregulated *Tardbp* mRNA levels. By contrast, the p.F210I loss-of-function mice did not display a motor phenotype when aged up to 2 years [108].

The causes of TDP-43 translocation out of the nucleus are unknown but may include genetic (mutation) or environmental factors. A p.M337V transgenic pig model of ALS that exhibits a severe phenotype was used to investigate the role of cytoplasmic mislocalization on RNA splicing events [109]. TDP-43 was observed to be cytoplasmic in neuronal cells and caused mislocalization of splicing factor proline-glutamine rich (PSF), and NeuN (neuronal nuclei), which regulates splicing of RNA. Expression of the mutant TDP-43 led to the increased production of an exclusion isoform of NMHC II-B (myh 10), which is important for the regulation of N-methyl-d-aspartate receptor trafficking and neuronal function and is regulated by PSF and NeuN. Protein analysis confirmed that there was decreased NMHC-II B. Importantly, mislocalization of PSF and NeuN to the cytoplasm was also observed in ALS patient brain tissue [109]. In a homozygous knockin mouse of TDP-43^M337V^, which did not show a motor phenotype, mRNA splicing was deregulated, including that of *Kcinp2*, *Sort1*, *Sema3f* [110]. Thus, environmental or genetic factors that induce TDP-43 cytoplasmic mislocalization may contribute to ALS and FTLD pathogenesis via altered mRNA splicing.

TDP-43 deficiency in mouse embryonic stem cells decreased the splicing of non-conserved cryptic exons, leading to the incorporation of these exons into the mRNAs, translation and subsequent nonsense-mediated decay. Reduced TDP-43 repression of these exons was also observed in cases of ALS and FTLD [111]. The authors fused the N-terminus of TDP-43 to a well-characterized splicing repressor domain to determine that TDP-43 repression of these exons was important for cell viability [111]. In drosophila, null mutants of TBPH (TDP-43 homologue) led to altered transcript splicing, including that of a *cacophony*, a voltage-gated calcium channel. This affected neuromuscular junctions. Of note, expression of cacophony rescued the locomotion phenotype indicating that changes to splicing due to TDP-43 are functionally significant in ALS [112].

##### RNA Binding and Regulation of Gene Expression

TDP-43 promotes mRNA instability by interacting with Caf1, and thereby facilitates RNA deadenylation, which is the rate-limiting step of mRNA decay [113]. Depletion of TDP-43 by lentiviral shRNA in embryonic mouse motor neurons leads to defects in axon outgrowth along with transcriptional changes (118 upregulated and 136 downregulated transcripts), impaired protein synthesis and mitochondrial function in axonal subcellular compartments [114]. The mRNAs that were downregulated were shown to have TDP-43-binding sites in the 3′ UTRs, where those that were upregulated had fewer interaction sites in their 3′ UTRs [114]. Expression of a chimeric repressor consisting of the TDP-43 RRM domain fused with an unrelated splicing repressor (Raver1) reduced the motor defect of a conditional motor neuron TDP-43 knockout mouse, extending lifespan [101]. This suggests that TDP-43-mediated repression of mRNA translation may be dysfunctional in ALS and FTLD. Using patient-derived cells from sporadic ALS- and *C9orf72* repeat-positive ALS cases, Tank et al. (2018) observed a severe destabilization of RNA transcripts. This destabilization was mirrored by overexpression of TDP-43. The authors show that energy production may be impeded, while protein synthesis was increased, potentially as compensatory mechanisms. Reduced transcripts for oxidative phosphorylation genes and ribosome protein-coding RNAs were also observed in the spinal cord from ALS patients [104]. Recognition motifs for RBPs, including TDP-43, were enriched among the RNAs that had altered stability, indicating that RBPs may be important for pathogenesis [104]. Using chromatin isolation by RNA purification and omics strategies, TDP-43 was found to bind to the neuron-specific long-non-coding RNA neuroLNC [115]. TDP-43 was further required for critical processes in neuronal development and promoted the stabilization of selected mRNAs important for synaptic processes. Blocking the interaction between neuroLNC and TDP-43 abolished the exocytotic release of neurotransmitters at the synapse that is mediated by neuroLNC [115].

Expression of p.Q331K or p.M337V mutant TDP-43 in mice leads to a motor phenotype [106,116] and may be related to changes to the translatome of motor neurons [117]. Expression of TDP-43^A315T^ in mice led to upregulation of SYNGR4 and downregulation of PLEKHB1. These changes were also observed in an additional mouse model expressing TDP-43^Q331K^ [117]. The functions of these proteins in motor neurons are unknown but may involve modulation of vesicular protein transport (via *SYNGR4*) or impact G protein-coupled signaling and protein trafficking via interaction with myosin (Pleckstrin homology domain-containing protein family B member 1) [117]. In NSC-34 cells and primary cortical neurons, overexpression of either wild-type or p.A315T enhanced translation of *Camta1*, *Mig12* and *Dennd4a* mRNAs. Camta1 and Dennd4a are associated with neurodegeneration, thus TDP-43 regulation of these transcripts may be involved in ALS and FTLD pathogenesis [118].

TDP-43 binds to the 3′ untranslated region to promote instability of mRNA transcripts of tau, vascular endothelial growth factor A and progranulin, all implicated in ALS and/or FTLD [91,119,120]. Another target mRNA of TDP-43 is the autophagy regulator *Atg7.* A mutant TDP-43 lacking the RRM1 domain had impaired ability to bind to and stabilize Atg7 mRNA, leading to impaired autophagy [121]. TDP-43 also represses cryptic isoforms of Atg4 [111]. A further autophagy-related target of TDP-43 is raptor [122]. Thus, TDP-43 appears to be important for autophagy regulation (see Section 2.4.5.). However, the mRNA with the greatest increase in binding in FTLD-TDP brain tissue compared with controls was to the non-coding RNAs nuclear paraspeckle assembly transcript 1 (NEAT1) and metastasis-associated lung adenocarcinoma transcript 1 (MALAT1; also known as NEAT2) [123]. Decreased binding was observed for the neurexin 3 and glial excitatory amino acid transporter 2 (EEAT2) transcripts. EEAT2 is involved in the clearance of glutamate at the synapse. The majority of altered cDNAs identified mapped to introns, thus the authors speculated that TDP-43 might be regulating splicing of these mRNAs. Indeed, following knockdown of TDP-43 they found alternative splicing including in those mRNAs that are important for neuronal development and survival [123].

TDP-43 and has a regulatory role in gene expression and silencing and the stress response via interaction with G-quadruplex (G4)-RNA. G4 is a structure found in DNA or RNA that are rich in guanines. The structure of G4 is important for long-distance transport and translation of mRNA in neurons. Ishiguro et al. (2020) investigated the effects of 10 randomly selected ALS mutants (p.D169G, p.N267S, p.G287S, p.G295S, p.A315T, p.G348C, p.P363A, p.S379P, p.N390D and p.D247A) on interaction with G4-RNA in HEK293 cells. All mutant proteins showed reduced G4-RNA binding and decreased trafficking of G4-RNA [124]. As all mutations tested were located within the glycine-rich region, this may be crucial for TDP-43 binding to G4-RNA and RNA transport. A small molecule that displaces G4-RNA from TDP-43 complexes prevented toxicity in a drosophila TDP-43 ALS model, suggesting that the G4 structure may be important for pathogenesis [107].

### 2.4. Gain of Toxic Cytoplasmic Function

#### 2.4.1. Stress Granules

Stress granules are protective cytoplasmic granules that form in response to cellular insults to protect and sequester non-essential mRNA transcripts (117Stress granules sequester stabilized mRNA transcripts that are not required for a cellular defense mechanism, therefore establishing a cellular transcript bias promoting cellular survival factors [125]. Stress granules are observed following heat shock, osmotic stress, oxidative stress, nutrient starvation, endoplasmic reticulum stress, UV radiation and viral infection [125,126,127]. In response to stress, signaling pathways are initiated to alter gene expression by either transcriptional alterations or through post-translational modifications that alter RNA-binding proteins [128]. Stress granule composition consists of RNA-binding proteins, polyA-binding proteins, 40S ribosomal subunit, protected mRNAs and eukaryotic initiation factors [129]. RNA-binding proteins, such as TDP-43, are integral to the formation and regulation of stress granules [120]. TDP-43 has been identified in stress granules in vivo and functions to regulate stress granule assembly and disassembly [68,108,120]. Stress granules present a probable precursor to TDP-43 proteinopathies observed in cases of ALS and FTLD [119]. The initiation of stress granule formation is coupled to the phosphorylation of eukaryotic initiation factor 2 alpha (eIF2α) [125]. Phosphorylation of eIF2α disrupts the ribosomal formation around translating mRNA transcripts by blocking the formation of the initiation complex eIF2α-GTP-tRNA^met^. Disrupting the initiation complex from binding to ribosomes permits the binding of T-cell intracellular antigen 1 (TIA-1) to the 48S ribosomal complex [125]. TIA-1 binding to the ribosomal subunit promotes its disassembly and subsequent assembly of stress granules [128]. TIA-1 and RasGAP-association endoribonuclease (G3BP) are integral stress granule proteins. G3BP is a nucleator of stress granules involved in assembly [130]. TDP-43 regulates both TIA-1 and G3BP at the mRNA level as silencing TDP-43 promotes the expression of TIA-1 by 130% while reducing G3BP expression by 79% [128]. Additionally, siRNA knockdown of TDP-43 decreased the size of stress granules and decreased the rate of stress granule assembly. As such, cells lacking TDP-43 demonstrated a greater sensitivity to stress [128].

RNA-binding proteins are integral to the function and maintenance of stress granules. TDP-43 is considered an important regulator of stress granule assembly, disassembly and maintenance. Using a global proteomics approach in an over-expression model of FLAG-tagged TDP-43 in HEK293 cells, an interactome of 126 proteins was identified that uniquely bound to TDP-43. Of these, 15 proteins are associated with stress granules [102]. Studies performed in HeLa cells demonstrate that TDP-43 co-localizes with stress granule markers during oxidative stress [128]. Studies in patient lymphoblast cells expressing TDP-43^WT^, TDP-43^D169G^ or TDP-43^R316S^ demonstrated that following sodium arsenate treatment, the mutant TDP-43 were more resistant to stress granule disassembly [128]. Therefore, TDP-43 assists in both the assembly and disassembly of stress granules during times of stress. Further mutational studies of TDP-43 reveal conflicting results regarding the role of TDP-43 in stress granule dynamics. Mutants p.A315T and p.Q343R are increased in the insoluble fraction of cells, which also contained stress granule markers such as TIA-1 [131]. Mutant p.M337V displays increased cytoplasmic mislocalization, but had a reduced association with TIA-1 and reduced the number of cells containing stress granules. Thus, TDP-43 mislocalization into the cytoplasm and altered stress granule dynamics may be intrinsically linked [116]. When expressed in patients’ primary fibroblasts the p.A315T mutant protein did not translocate to stress granules. However, expression of TDP-43^A315T^ resulted in the downregulation of G3BP by 78% [130]. Thus, a loss of G3BP may be responsible for stress granule assembly difficulties in cells expressing mutant TDP-43. The most prevalent TDP-43 mutation, p.G348C, inhibits the formation of stress granules. Furthermore, TDP-43-ΔNLS proteins impaired stress granule formation revealing the possibility that reduced nuclear TDP-43 in ALS and FTLD may impair stress granule formation [75]. A screen of over 9000 compounds identified small molecules that impair TDP-43 binding to RNA and recruitment to stress granules, while extending survival in a TDP-43 overexpression mouse model of ALS [132]. Thus, impaired stress granule formation may be critical to ALS pathogenesis.

#### 2.4.2. Phase Separation

Liquid–liquid phase separation (LLPS) is the phenomenon that drives the formation of membrane-less organelles and protein aggregation [129]. The low complexity C-terminal domain of TDP-43 contributes to the LLPS of stress granules and cytoplasmic bodies observed in ALS/FTLD patients [126]. Liquid–liquid phase separation of RBPs drives the formation of liquid droplet-like RNP granules that lack a membrane. Stress induced by arsenic treatment leads to the formation of liquid droplet-like nuclear bodies that are dynamic and reversible and protected against cytotoxicity [73]. Granules formed in SH-SY5Y cells demonstrated both cytoplasmic and nuclear TDP-43 bodies that were 0.2–1 μM in size. TDP-43 overexpression increased granule size to 1–3 μM [133]. The axonal localization of TDP-43 granules in rat primary cortical neurons alters granule dynamics [95]. TDP-43-positive granules located in the proximal axon are less dynamic and more stable compared to mid axonal granules. The differences in granule dynamics may arise through maturation of the oligomerized granule into a more fibrillated state. The proximal localization of granules may confer the possibility of granule aging/persistence as a component in the progression of ALS. Additionally, proximal localization of TDP-43 aggregates confers a greater resistance to chemical modulation by 1,6-hexanediol [95]. Of note, ALS-linked mutants p.M337V and p.G296S disrupt TDP-43 granule dynamics. Mutant granules displayed less efficient transport and incomplete recovery. The C-terminal mutant TDP-43 containing granules display a 20-fold increased viscosity and had greater resistance to 1,6-hexanediol treatment, thereby demonstrating greater internal stability [95].

The liquid–liquid phase separation of TDP-43 is regulated through the low complexity domains. The C-terminal domain of TDP-43 is a 160-residue domain that contains very low sequence complexity that contains only a single alpha-helix between residues 321 and 330 [134]. The majority of disease-causing mutations are identified in this C-terminal domain of TDP-43. Residues 331–340 regulate the helix structure with Trp334 initiating the folding of the secondary structure [135]. Glycine residues at residue positions 335 and 338 are inhibitors of helix formation outlining potential regulatory elements. The ALS-linked mutant p.G335D contained enhanced alpha helical structures [134]. The helical structure inside the C-terminal region of TDP-43 is critical for the formation of granules mediated by LLPS. Fragmented TDP-43 (CTFs) is observed in ALS patients. An intact C-terminal domain reduces solubility and affect phase separation into cytoplasmic granules or their disassembly [134]. Several ALS-linked mutants including p.M337V, p.A321G, p.A321V and p.Q331K disrupt the helix interaction inside the C-terminal domain. This disruption results in the increased propensity of TDP-43 to phase separate and lead to insoluble aggregates [134].

Phase separation and the formation of stress granules present a possible precursor to cytoplasmic aggregates of TDP-43 [136]. TDP-43 granules co-localize to known stress granule proteins such as fused in sarcoma/translocated in liposarcoma, human ribonuclear protein A1 and TIA-1. In addition, arsenic-induced stress promotes the formation of TDP-43 immobile bodies and demonstrates a connection between stress granules and TDP-43 aggregation [137]. However, there is evidence suggesting that TDP-43 granules co-localize either dependently or independently of stress granule markers [138]. The recruitment of TDP-43 into stress granules is correlated to the types of stress encountered by the cell. Following acute stress, TDP-43 is not recruited to stress granules [138]. With chronic stress, however, TDP-43 is recruited to stress granules regardless of the ALS-linked mutation, suggesting that TDP-43 immobile bodies arise from prolonged stress through impaired stress granule disassembly. Therefore, it is speculated that during neurodegeneration, a critical threshold exists between stress granule disassembly and TDP-43 aggregation [137]. The presence or absence of RNA alters the formation and dynamics of TDP-43 granules. TDP-43 binds to RNA through UG-rich sequences and these complexes are present within phase separated structures. The presence of RNA inhibits the homo-oligomerization of TDP-43 in granules. An optogenetic approach using a Cry2-TDP-43 construct demonstrated that light-induced, phase-separated TDP-43 was void of RNA [136]. Alternatively, TDP-43 was shown to localize with NEAT1 and upregulation of *NEAT1* promoted the LLPS of TDP-43 [73]. Importantly, the ALS-associated p.D169G mutation impaired the assembly of TDP-43 into NEAT-1-positive nuclear bodies, instead p.D169G translocated to the cytoplasm and formed additional stress granules than were observed for wild-type TDP-43. However, the ALS-associated mutant proteins p.Q331K and p.M337V did not affect nuclear body formation [73]. Thus, while TDP-43 is important for reducing the toxicity of stress via the formation of reversible nuclear bodies, whether this process is impaired in ALS and FTLD remains to be determined. An endogenous mouse mutation (p.M323K) and the ALS-associated p.Q331K mutation decreased the ability of the mutant proteins to phase separate compared with wild-type [108]. Li et al. (2018) identified key Tryptophan residues (Trp-334, Trp-385 and Trp-412) that are critical for LLPS, and suggest that the ~50 mutations that manifest in the C-terminal domain may interfere with the motifs required for LLPS to occur [135].

#### 2.4.3. Impaired Axonal Transport

Axonal transport functions to deliver and distribute cellular growth factors, organelles, synaptic cargoes and cytoskeletal factors to increase the efficiency of trafficking within neuronal cells [139]. Impaired axonal transport is observed in mouse models and post-mortem tissues of patients with ALS [140]. Mutant TDP-43 proteins demonstrate impaired anterograde movement within neurons, which is likely to play a role in neuron axon growth [29]. Axonal transport is critical to neuron function and survival [141]. Abnormal mitochondrial trafficking and abnormal vesicles in the axon have been identified in ALS [142,143]. TDP-43 is highly mobile and is actively transported in motor neuron axons, with the level of axonal TDP-43 increased by treatment with brain derived neurotrophic factor. Overexpression of either wild-type (full-length) or mutant (p.M337V or p.A382T) TDP-43 in primary motor neurons impaired axon outgrowth. Both mutant proteins were more abundant in the axons than wild-type TDP-43 [29]. However, mutant TDP-43 expression (p. M337V or p. Q331K) leads to impaired axonal transport in rat cortical neurons [25]. Further, the microtubule-dependent transport of TDP-43 mRNA granules is suppressed by ALS-associated mutations (p.M337V or p.A315T) [21]. Transport of mitochondria is also impaired by overexpression of either wild-type or mutant (p.Q331K, p.A315T or p.M337V) TDP-43 [144,145]. Wild-type TDP-43 is observed to move bidirectionally within neurons for long distances with brief pauses and displays a greater association with anterograde movement towards the NMJ. Mutant TDP-43 (p.M337V and p.A315T) impaired the anterograde movement of TDP-43 in neurons and altered the localization pattern of TDP-43 away from the NMJ and proximal axons. Similar results were observed in mouse primary cortical neurons. Treatment with nocodazole revealed that axonal transport of TDP-43 is microtubule-dependent. [21]. Thus, TDP-43 appears to play a role in axon growth that may be dependent on its axonal transport and appears to be impaired by ALS or FTLD-associated mutations.

#### 2.4.4. Mitochondrial Dysfunction

Mitochondrial dysfunction has been implicated in the disease progression since the observation of abnormal mitochondrial morphology and the identification of *SOD1* mutations in ALS patients [136]. SOD1 is involved in the breakdown of reactive oxygen species (ROS) within the cell [146]. Reactive oxygen species are a byproduct of the mitochondrial electron transport chain. Therefore, ALS was linked with dysfunctional mitochondria through oxidative stress [146]. ALS patient tissues display abnormal mitochondrial morphology [147]. As neurons have a particularly high demand for mitochondria due to synaptic homeostasis, mitochondrial dysfunction in neurons has a significant effect on function and survival [148]. The breakthrough discovery of TDP-43 as a pathological factor in ALS in 2006 does not exclude mitochondrial involvement in the disease pathology of ALS. TDP-43 links mitochondrial dysfunction to ALS and FTLD through its regulation of nuclear-encoded mitochondrial genes [143]. Mitochondrial caspases cleave TDP-43 into the pathological 25 and 35 kDa fragments associated with ALS and FTLD, thereby linking mitochondrial defects and TDP-43 to disease proteinopathy [146]. 

##### TDP-43 and Mitochondrial mRNA Regulation

There is growing evidence that early mitochondrial dysfunction within upper motor neurons leads to their vulnerability to TDP-43 proteinopathy [145]. TDP-43 is at least partially localized to mitochondria within the diseased spinal cord and frontal cortex tissues of patient samples [149]. Further, TDP-43 p.A315T mutant mice display mitochondrial defects early in life that are restricted to motor neurons [150]. TDP-43 binds to micro RNA (miRNA) and regulates mitochondrial mRNAs. Silencing of TDP-43 through siRNA demonstrated that 13.01% of upregulated proteins were involved in mitochondrial processes. TDP-43 is involved in miRNA regulation as it has been colocalized to both Drosha and Dicer, but not Ago2. Drosha and Dicer are involved in the production of miRNAs, while Ago2 is involved in silencing via binding and trafficking of miRNAs [146]. ALS-linked mutants enhance miRNA trapping and subsequently increase the expression of mitochondria-bound proteins. These differentially regulated proteins include those involved in oxidative phosphorylation and transport porins. The mitochondrial protein imbalance generates additional ROS and TDP-43 cleavage causing subsequent TDP-43 aggregation [146]. TDP-43 also stabilizes the polycistronic RNA transcripts of proteins involved in the electron transport chain, tRNAs and mitochondrial ribosomal RNAs [151]. Izumikawa et al. (2017) generated single point mutations (K136, K145 and F147/149) to demonstrate that the RRM1 domain is critical for regulating tRNA interaction in mitochondria. They further showed that TDP-43 overexpression alters mitochondrial morphology and inhibits cell proliferation [151]. Treatment with ethidium bromide to inhibit mitochondrial transcription reduced the overexpression effects of TDP-43 on cell proliferation. Therefore, TDP-43 regulation of mitochondrial transcripts has global cellular effects [151]. 

##### Mitochondrial Structural and Functional Abnormalities

Electron microscopy of patient samples revealed mitochondrial impairment. Similar impairments, including the presence of smaller mitochondria with swollen and degenerated cristae, or the complete lack of cristae, were observed in cell and animal models of overexpressed TDP-43^A315T^ [152]. This was shown to a lesser extent in models with wild-type TDP-43 overexpression. These degenerative changes were accompanied by reduced membrane potential, increased ROS, reduced mitochondrial ATP and activation of the mitochondrial unfolded protein response (UPR). The authors show that mitochondrial impairments occur early and precede cell death [152]. In a cell line stably expressing a 25 kDa CTF, markers for mitochondrial oxidative stress were increased compared to EGFP-expressing cells. In addition, the cells had swollen mitochondria with dilated cristae [153]. In mice expressing TDP-43^A315T^ under the prion promoter, mitochondrial defects are observed as early as postnatal day 15 indicating that they may contribute to motor neuron vulnerability [150]. The mitochondria were distorted with compromised inner membranes or broken outer membranes, or formed ring-like structures [150]. In cells overexpressing wild-type, mutant (p.Q331K or p.M337V) or CTFs (TDP-25 or TDP-35), the percentage of cells with abnormal mitochondria was increased compared to empty vector transfected cells [154]. The number of mitochondria was reduced in cells transfected with TDP-25 or TDP-35, which localized to mitochondria. Further, expression of CTFs was associated with upregulation of the autophagy marker LC3-II and reduced the expression of p62, changes that indicate that CTFs induce mitophagy [154]. 

Overexpression of wild-type TDP-43 decreased the length and density of mitochondria in primary motor neurons. This was enhanced in cells expressing mutant (p.Q331K or p.M337V) TDP-43 [145]. Importantly, while disease mutations (p.Q331K and p.M337V) increase the co-localization of TDP-43 with mitochondria, inhibition of mitochondrial localization decreases TDP-43-mediated neurotoxicity [149]. Both suppression and overexpression of TDP-43 decreased the movement of mitochondria. Expression of mutant TDP-43 led to reduced mitochondrial length [149]. Wang et al. (2017) showed that the motor phenotype developed by 8 months of age in hemizygous TDP-43^M337V^ mice could be alleviated with the use of an inhibitor that prevented TDP-43 localization to mitochondria [155]. The interaction between TDP-43 and the mitochondria appears to be mediated through vamp-associated protein b and c [156]. In addition to, direct interaction with mitochondria affecting mitochondrial morphology and trafficking, TDP-43 also regulates the expression of genes that are involved in mitophagy. Parkin is an E3 ubiquitin ligase that in co-ordination with PTEN-induced putative kinase 1 (PINK1) is involved in mitochondrial degradation via mitophagy [157]. Overexpression of TDP-43 decreased *Parkin* transcripts, but not that of *PINK1* [145]. However, there was a significant increase in the amount of insoluble, cleaved PINK1 observed in Western blots and TDP-43 overexpression reduced the co-localization of PINK1 to mitochondria. Further, endogenous PINK1 accumulates in the neurons of TDP-43^Q331K^ mice. The increase in cleaved PINK1 caused mitochondrial dysfunction [157]. 

Mitochondrial involvement in the progression of ALS may be mediated via apoptotic signals in calcium buffering and the activation of caspases. Calcium signaling is an essential mechanism involved in normal neuronal function [158]. Signal transduction involves the uptake and release of calcium stores to initiate electrical signaling between synapses. C9orf72 GGGGCC_n_ repeat sequence expansions, the most common cause of ALS and FLTD, impairs mitochondria and the calcium balance within neurons. Low calcium levels correlate with a low response in motor neurons through depolarization, decreasing recovery times and neuronal function [158]. In *SOD1* mutant neurons in addition to a disruption of cellular calcium stores, impaired mitochondria generate activated caspase-1, -3, -7 and -9. This demonstrated a link between mitochondrial imbalance and ALS [159]. Like SOD1 mutant proteins, mutant TDP-43 (p.M337V and p.I383T) also impair calcium uptake by mitochondria [158]. Thus, TDP-43 (wild-type and mutant) may affect mitochondrial structure and function through multiple mechanisms, including the production of oxygen radicals.

##### Oxidative Stress

Reactive oxygen species (ROS) are produced due to electron leakage that occurs during oxidative phosphorylation within the mitochondrial energy production pathway. Irregularities in the regulation of ROS and reactive nitrogen species (RNS) can cause oxidative stress. Oxidative stress can be defined as the imbalance between the production and the clearance of ROS/RNS that may result in molecular damage [160]. Oxidative stress has been suggested to increase with age, and harm mRNA sequences involved in protein synthesis, and the mitochondrial electron transport chain, inducing the production of impaired proteins. Oxidative stress is associated with mitochondrial pathways energy deficits, calcium imbalance and apoptotic mechanisms, all of which occur in ALS [160].

The cytoplasmic mislocalization and aggregation of TDP-43 has been associated with oxidative stress [61]. Irregular RNA processing occurs under oxidative conditions [79]. Cohen et al. (2012) used neuronal tissue in vitro to show that oxidative stress promotes insolubility via the cross-linking of TDP-43 which is induced by cysteine oxidation and disulphide bond formation. Increases in the amount of cross-linked TDP-43 was observed in ALS and FTD-TDP brain samples, pointing to TDP-43 cross linking as a pathological feature in these diseases [61]. In their investigation, oxidative stressors such as hydrogen peroxide, arsenic and heat shock all resulted in TDP-43 insolubility. Hydrogen peroxide resulted in TDP-43 mislocalization from the nucleus into stress granules in the cytoplasm, whilst arsenic resulted in TDP-43 aggregates within the nucleus, both showed similar insoluble cross-linked TDP-43 variants [61]. Oxidative stress may contribute to the pathophysiology of ALS by acting as a trigger for stress granule formation [161]. Expression of wild-type or mutant (p.Q331K or p.M337V) TDP-43 decreased membrane potential (∆Ψm) and was correlated with increased ROS production. CTFs TDP-25 or TDP-35 also increased ROS production [154]. In SH-SY5Y cells expressing TDP-43^Q331K^, increased levels of ROS were observed, along with increased DNA strand breaks and apoptosis [162].

The functional connectivity of motor neurons has been investigated in an oxidative environment (induced by arsenic) using embryonic stem cells from mouse models expressing either wild-type or p.M337V TDP-43 [163]. In comparison to cells expressing wild-type TDP-43, those carrying the p.M337V mutation demonstrated reduced recruitment of proteins involved in ER and endosomal-extracellular transport pathways. In response to oxidative stress, reduced binding of mutant TDP-43^M337V^ to the cytoplasmic poly (A)-binding protein and eukaryotic initiation factor 4A-I, essential proteins for initiation of stress granule formation, was observed and may be linked to the impaired stress granule formation that is observed in the TDP-43^M337V^ motor neurons [163]. Thus, oxidative stress may cause irregular activity of TDP-43-mediated pathways, with the mutations leading to impaired extracellular vesicle secretion in motor neurons [163].

#### 2.4.5. Proteostasis

Proteostasis refers to the maintenance of protein homeostasis and balances the production of nascently translated proteins with the degradation of misfolded or aggregated proteins [164]. Protein quality control ensures that newly translated proteins are folded correctly. For proteins that will be secreted or are destined for the plasma or cell membranes, this occurs in the endoplasmic reticulum [165]. If a protein cannot be refolded into the correct configuration by molecular chaperones, such as the heat shock proteins, it will be delivered to the UPS for degradation, whereas aggregated, ubiquitinated proteins are shuttled to the autophagy-lysosome system (autophagy) for degradation [164]. Aberrant protein aggregation is a characteristic of neurodegenerative disorders including ALS and FTLD, such aggregates normally constitute proteins that are misfolded and may indicate an impairment to proteostasis mechanisms [7,166].

##### ER Stress and the Unfolded Protein Response

ER stress is a characteristic of ALS and FTLD [167,168]. When unfolded proteins accumulate in the ER, termed ER stress, this activates various signaling pathways, termed the UPR [169]. If ER stress is not resolved, cell death may occur [170]. Large amounts of calcium (Ca^2+^) are stored in the ER. It plays a role in the ER mitochondrial calcium cycle, which couples mitochondrial energy production and ER protein processing with neuronal synaptic activity [167]. Interaction between the ER membrane and mitochondria is important for metabolomic processes such as energy production, protein folding, autophagy, apoptosis and the biogenesis and transport of mitochondria [156]. However, the mechanisms that tie the ER to mitochondria are not completely understood. TDP-43 has been shown to interrupt an interaction between the ER protein vamp-associated protein b and c and the mitochondria localized protein tyrosine phosphatase interacting protein 51, which together regulate the association between the ER and mitochondria [156]. Overexpression of wild-type or mutant (p.M337V, p.Q331K, p.A382T, p.G348C) TDP-43 interrupts this ER-mitochondria interaction, possibly via decreased interaction with VAPB. Expression of TDP-43 was also associated with the activation of glycogen synthase kinase 3β (GSK-3β) and disturbed Ca^2+^ homeostasis. Activation of GSK-3β and disrupted Ca^2+^ homeostasis are observed in ALS [156]. In cell lines expressing TDP-43 p.A382T or p.M337V, Ca^2+^ signaling from the ER was reduced 50% compared with cells expressing wild-type TDP-43 [171]. Decreasing the Ca^2+^ in wild-type TDP-43-expressing cells mimicked the cellular phenotype of mutant TDP-43 expressing cells, indicating that TDP-43 pathology may be linked with reduced Ca^2+^ [171]. In small animal models of TDP-43 proteinopathy (worms and zebrafish), pharmacological reduction in ER stress was neuroprotective against TDP-43 proteinopathy induced by overexpression of TDP-43^G348C^ [170]. Expression of TDP-43^A315T^ in SH-SY5Y cells increased cytotoxicity by activating ER stress-induced autophagy, with cell death reduced by autophagy inhibition [172]. Thus, targeting the ER-UPR may be an important avenue for research into new therapeutics for ALS and FTLD.

##### Ubiquitin-Proteasome System

If the ER is unable to refold a misfolded protein, it is shuttled to the UPS for degradation [169]. The UPS is a protein degradation system in which ubiquitin is activated and transferred to ubiquitin conjugases that then bind the ubiquitin to target substrates with the assistance of ubiquitin ligases. Ubiquitinated proteins are then transported to a proteasome for degradation [173]. In neurodegenerative diseases such as ALS, UPS function may be compromised, resulting in inappropriate protein degradation and protein accumulation, which can lead to apoptosis [174]. The level of TDP-43 in neurons is critical, as both overexpression and knockdown of the protein in various animal models causes neurodegeneration [37]. Overexpression of TDP-43 leads to a cellular phenotype that is reminiscent of ALS and FTLD TDP-43 proteinopathies. This includes increased insoluble polyubiquitinated proteins due to impaired proteasome function [175]. In neuronal cells stably expressing TDP-25, cytotoxicity was dependent on the proteasome [153]. Of note the cytotoxicity of overexpressed TDP-43 and that of proteasome inhibition is mediated by phosphorylation of the ubiquitin-binding domain of the autophagy cargo receptor p62 by tank-binding kinase 1 (TBK1) [175], which is mutated in up to 4% of ALS and FTLD cases and up to 10.8% of FTLD-ALS cases [176]. Recently, reduced phosphorylation of p62 by a TBK1 ALS-associated mutant protein was linked with decreased autophagic degradation of TDP-43, suggesting that TBK1 may be a novel regulator of TDP-43 levels [177]. 

TDP-43, either full-length or truncated forms, can be degraded via both the UPS and autophagy [48,178,179,180]. Scotter et al. (2014) showed that the pathway used for TDP-43 degradation is dependent on whether it is in soluble (UPS) or aggregated (autophagy) form [37]. Araki et al. (2014) performed pulse chase experiments in SH-SY5Y cells stably expressing TDP-43 mutant (p. G298S and p.A382T) proteins [181]. They showed that these TDP-43 mutant proteins had an increased turnover compared with wild-type TDP-43, which was partially prevented by a proteasome inhibitor, but not a lysosomal inhibitor. Thus, these TDP-43 mutant proteins are degraded by the UPS [181]. Of note, inhibition of the proteasome, but not other stress conditions, induced the phosphorylation, ubiquitination and cytoplasmic aggregation of TDP-43, epitomizing ALS and FTLD TDP-43 proteinopathies [182]. Additionally, motor neuron specific genetic disruption of the proteasome, but not of autophagy, in mice led to the development of an ALS phenotype [183]. Thus, impairment of the UPS may be critical to ALS/FTLD pathology. 

##### Autophagy

Dysfunctional autophagy is often observed in neurodegenerative disorders. Autophagy is a cellular mechanism for the removal of aggregated proteins and damaged organelles, via selective or non-selective (bulk) processes. It can be divided into microautophagy, macroautophagy or chaperone-mediated autophagy [7]. Unless otherwise specified, the following information refers to macroautophagy, hereafter referred to as autophagy. Neurons are uniquely susceptible to impairments in the autophagy-lysosome system. This in part is due to being non-divisible and therefore unable to dilute toxic protein aggregates. Additionally, while autophagosomes arise throughout the neuron, the lysosomes that are required for proteolysis are mostly located within the soma, therefore the autophagosome must be actively transported [7]. Thus, impairments to autophagosome formation or maturation, impaired fusion with lysosomes, defects in transport or lysosomal degradation have the potential to impact neurons greatly.

While TDP-43 aggregation and autophagy dysregulation have been highlighted in ALS and FTLD pathology for some time, the role of TDP-43 in autophagy regulation was unknown. However, knockdown of TDP-43 led to decreased expression of the critical autophagy gene *Atg7* [121]. This caused the accumulation of polyubiquitinated proteins and the autophagy cargo receptor p62, suggesting that TDP-43 may form part of an autophagy feedback regulatory loop that is potentially disrupted in TDP-43 proteinopathies [121]. A role for TDP-43 in autophagy regulation was further demonstrated in mice homozygous for a TDP-25 transgene, which displayed reduced induction of autophagy and impaired proteasome function [184]. The mice showed a gene-dosage effect, as the homozygous mice had decreased cognition and reduced motor function compared with heterozygotes [184]. Autophagy was also inhibited in a knockin TDP-43^N390D^ mouse model. This was due to a progressive increase in TDP-43 levels, which upregulated autophagy regulator *Bcl-2*, with the mouse showing an ALS phenotype [36]. TDP-43 knockdown targeted the mammalian target of rapamycin (mTOR) complex 1 component raptor, which in turn induced the nuclear localization of transcription factor EB (TFEB) causing transcriptional changes to genes that increase the biogenesis of autophagosomes and lysosomes [122]. However, TDP-43 knockdown impaired autophagosome-lysosome fusion due to a downregulation of dynactin 1. The effects of TDP-43 knockdown on autophagy were shown to be mTOR-dependent (TFEB regulation) and mTOR-independent (fusion) [122]. A state of increased autophagy induction paired with an accumulation of immature or intermediatory autophagosomes occurs in neurodegenerative conditions, including Alzheimer’s disease, and leads to overwhelm of the autophagy-lysosome system [185]. In ALS and FTLD, TDP-43 loss of function due to cytoplasmic mislocalization and/or aggregation may partly explain the impairments in autophagosome-lysosome fusion and autophagy-mediated protein degradation observed. 

In immortalized motor neurons and stabilized myoblasts the overexpression of TDP-25 resulted in impaired autophagy and TDP-25 was instead cleared by the proteasome [186]. In a Neura2A overexpression model, TDP-25 formed insoluble cytoplasmic aggregates within 6 h of transfection. The amount of TDP-25 present in the insoluble fraction was reduced by treatment with berberine, an herbal autophagy activator that works via the inhibition of mTOR. The effects of berberine on TDP-43 aggregation were reversed by an inhibitor of autophagosome formation (3-MA) [187]. In HEK293 cells overexpressing TDP-43, the autophagy activator ibudilast increased the clearance of both TDP-25 aggregates and the full-length TDP-43 aggregates that formed in response to proteasome inhibition. The treatment reduced the cytotoxicity associated with TDP-25 overexpression and similarly to berberine worked via mTOR inhibition [188]. In an SH-SY5Y overexpression model, treatment with the mTOR-independent inducer of autophagy trehalose caused TDP-43 clearance via activation of TFEB. Trehalose has shown promise as a neuroprotective agent in various neurodegenerative diseases [189]. In an FTLD-U mouse model that exhibits TDP-43 proteinopathy due to overexpression of wild-type TDP-43, treatment with various autophagy activators (rapamycin, spermidine, carbamepine and tamoxifen) rescued motor dysfunction, reducing the level of TDP-43 inclusions and CTFs generated and alleviating caspase-mediated apoptosis [190]. Thus, autophagy inducers may be beneficial treatment options for ALS and FTLD patients.

### 2.5. Non-Cell-Autonomous Mechanisms

The expression of mutant TDP-43 within non-neuronal muscle cells indicated non-cell-autonomous effects of these proteins. Wächter et al. (2015) showed that expression of TDP-43 p.A315T within muscle cells had a degenerative effect on motor neuron cells [191]. Healthy motor neurons were co-cultured on top of transgenic wild-type TDP-43 or the TDP-43 p.A315T expressing muscle cells. In comparison to the controls the TDP-43^A315T^ motor neurons showed significantly reduced neurite outgrowth and fewer branching points [191]. Serio et al. (2013) investigated human iPSC-derived astrocytes harboring the p.M337V mutation and found no adverse effects on co-cultured wild-type TDP-43 or TDP-43 p.M337V neurons. However, mislocalization of TDP-43 from the nucleus to the cytoplasm and a decreased rate of survival compared to controls was observed in the TDP-43 p.M337V astrocytes. Thus, astrocytes harboring mutant TDP-43 exhibit cell-autonomous effects, but not non-cell-autonomous effects [192]. 

#### 2.5.1. Gliosis

Glial cells are three times more abundant than neurons and work to support neuronal function [193]. Gliosis is a defensive mechanism that occurs after astrocyte activation in reaction to brain or spinal cord injury. Astrocytes are essential glial cells that are involved in the oxidative stress response, scar formation and tissue repair [194]. Astrogliosis is a reactive form of gliosis in which there is aberrant proliferation of astrocytes in reaction to neuron destruction in the central nervous system [195]. Astrogliosis has been detected in ALS/FTLD TDP-43 mouse models including those expressing the human TDP-43 p.A315T and p.M337V proteins [196,197], overexpression of mouse TDP-43 in the forebrain [198] and overexpression of human TDP-43 in neurons [199]. Ke et al. (2015) generated an inducible mouse model of ALS that expresses TDP-43^A315T^. When doxycycline is added to the feed expression of the transgene is suppressed. Expression of TDP-43^A315T^ induced a neurodegenerative phenotype that included pronounced activation of astrocytes. Importantly, when the expression of the transgene was suppressed motor and behavioral deficits were improved [200]. In a detailed characterization of a distinct TDP-43^A315T^ mouse, Bargsted et al. (2017) observed a significant loss of motor neurons, axonal degeneration and activation of astrocytes and microglia. Primary astrocytes isolated from another transgenic hTDP-43^A315T^ mouse had impaired _L_-glutamine uptake and abnormal accumulation of ATP [201]. In addition, a significant increase in astrocyte activation was associated with motor neuron loss, suggesting that mutant TDP-43 may contribute to ALS via cell-autonomous and non-cell-autonomous mechanisms [201]. Expression of TDP-43^M337V^ in astrocytes in rats led to the activation of both astrocytes and microglia and motor neuron loss [202]. However, in a doxycycline repression mouse model expressing TDP-43-ΔNLS, only subtle effects on microglia were seen despite significant motor neuron loss [203]. Yet, when the expression of the transgene was repressed, microglia proliferated and cleared TDP-43, indicating a role for these cells in neuroprotection [203].

#### 2.5.2. Prion-Like Propagation

Prion diseases involve the propagation of infectious particles of proteinaceous origin, leading to neurodegenerative diseases [204]. The production of amyloid-like species may be important to propagation and is associated with many neurodegenerative disorders. TDP-43 can form amyloid-like species, and this may be mediated by the low complexity C-terminal domain that is often mutated in ALS or the RRM domains [205,206,207]. This low complexity domain may impart prion-like properties [204]. Of note, TDP-43 mutations (p.A315E and p.A315T) convert TDP-43 into insoluble, irreversible aggregates [207]. Application of recombinant full-length aggregates of TDP-43 to cells exogenously expressing TDP-43 induces intracellular aggregation of TDP-43, proving that TDP-43 can seed aggregation [208]. Self-templating of TDP-43 aggregation was observed when the insoluble protein fractions obtained from human ALS- or TDP-43-positive FTLD tissue samples were applied to neuronal cells overexpressing TDP-43 [209]. This finding has since been confirmed in various studies using stable cells, cells overexpressing TDP-43 and co-cultured cells [210,211,212]. A further study showed that TDP-43 seeds are show trans-synaptic spreading [213]. Thus, several studies implicate a prion-like mechanism for the spread of disease in ALS- and TDP-43-associated FTLD.

### 2.6. Other

#### AMPK Signaling

AMP-activated protein kinase (AMPK) is an energy metabolism and stress pathway regulator. AMPK senses imbalance between ATP and AMP in the cell, often caused by oxidative stress or nutrient deprivation, and responds by activating glucose uptake, and autophagy, while decreasing protein translation and glycogen synthesis [214]. Activation of AMPK was shown to be reduced in the central nervous system of TDP-43A315T mice [215]. During the presymptomatic stages of the disease, TDP-43A315T demonstrated a reduction of 60% and 80% in the spinal cord and brain tissue, respectively. Once TDP-43A315T mice were symptomatic, the activation of AMPK was reduced by 30% and 60% in the spinal cord and brain, respectively. Therefore, TDP-43 leads to reduced central nervous system AMPK activation, most potently in symptomatic ALS. By contrast, SOD1G93A patients showed over activated AMPK [215].

Expression of phosphatases, which dephosphorylate AMPK, was assessed in the TDP-43A315T mice. Protein phosphatase 2A (PP2A) was significantly increased in TDP-43A315T mice and correlated with reduced activation of AMPK. In NSC-34 cells expressing TDP-43Q331K and TDP-43M337V AMPK activation was reduced to 50% and 70%, respectively, when compared with cells expressing wild-type TDP-43 [215]. Concurrently, cells expressing TDP-43Q331K and TDP-43M337V showed 40% and 120% increased expression of PP2A, respectively. Confirming that TDP-43 caused AMPK inactivation via PP2A, the inhibition of PP2A resulted in AMPK activation in cells expressing either wild-type or mutant TDP-43. Therefore, targeting AMPK inactivation via PP2A inhibition may represent a model for therapeutics in patients with TDP-43 proteinopathies [215].

## 3. Discussion

**Figure 2:**
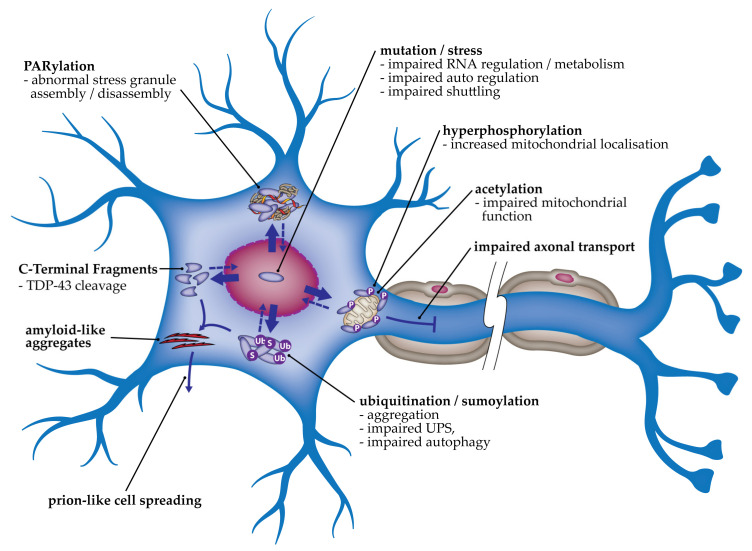
Molecular mechanisms underlying TDP-43 pathogenesis in response to mutations or stress. Mutations in *TARDBP* or other genes associated with ALS and FTLD, or exposure to oxidative stress or heavy metals such as arsenite, impair TDP-43 function. Specifically, TDP-43 autoregulation and nuclear:cytoplasmic shuttling is impaired, leading to a loss of RNA metabolism and increased cytoplasmic TDP-43. Mislocalization of TDP-43 increases its tendency to aggregate. Post-translational modifications occur including hyper-phosphorylation (P), acetylation, ubiquitination (Ub), SUMOylation (S), or PARylation, as well as cleavage/fragmentation, which promotes TDP-43 aggregation. The nuclear depletion, misfolding and cytoplasmic aggregation of TDP-43 leads to (1) dysregulation of RNA metabolism including splicing defects, (2) impaired mitochondrial function and axonal transport, (3) impaired proteostasis (UPS and autophagy), (4) abnormal stress granule dynamics and (5) amyloid-like aggregate formation that may be spread cell to cell in a prion-like manner.

TDP-43 is the primary pathogenic protein observed in almost all cases of ALS and the majority of non-tau-related FTLD. Expression of mutant proteins or overexpression of wild-type TDP-43 decreased axon outgrowth [29]. Mutant TDP-43 leads to denervation of neuromuscular junctions and decreased neuron viability [26,36]. However, the mechanisms underlying toxicity in cells expressing either mutant TDP-43 or overexpressing wild-type TDP-43 are incompletely understood. There is no overarching common disease mechanism that has been identified for all mutant proteins. It is possible that stress or mutations impair TDP-43 autoregulation, leading to increased expression of TDP-43. As TDP-43 is prone to aggregation, increased expression may promote misfolding or may prompt translocation into the cytoplasm where PTMs occur (Figure 2). Conversely, PTMs may promote misfolding and translocation. Further investigation is required to fully elucidate the mechanisms that lead to PTMs and TDP-43 cytoplasmic translocation. These modifications include hyper-phosphorylation, ubiquitination, SUMOlyation, acetylation and cleavage. It is critical to understand how translocation and modification are linked in order to understand the pathogenesis of these diseases. Further, the full consequences of these post-translational changes need to be elucidated to enable the development of effective treatments.

CTFs are observed in tissues from ALS and FTLD patients [16,18,45]. Cell models show that expression of various ALS-associated mutant proteins leads to the production of CTFs, which are hyperphosphorylated, aggregated and toxic [50,51,52,53,54]. Further studies show that cleavage and phosphorylation appear to occur post-aggregation [47]. It was thought that aggregation may be critical to the cytotoxicity of mutant proteins. However, overexpressed wild-type TDP-43 is toxic while tending not to aggregate [27]. Further, studies in transgenic mice show that it is translocation from the nucleus to the cytoplasm that is associated with a neuromuscular phenotype. In mice expressing TDP-43-ΔNLS neurodegeneration was observed despite the TDP-43-ΔNLS only occurring in cytoplasmic aggregates in a low proportion of cells [60]. Thus, the critical factor in pathogenesis appears to be TDP-43 translocation out of the nucleus. Many mutant proteins show increased cytoplasmic localization and/or decreased nuclear localization [25,28,56,72]. TDP-43 shuttles out of the nucleus through passive diffusion. However, it is unknown what causes TDP-43 translocation in ALS and FTLD. It is possible that in response to cellular stress or mutations, TDP-43 levels reach a critical point at which translocation occurs. This leads to a loss of function concerning TDP-43 regulation of RNA processing. Thus, while cytoplasmic aggregation of TDP-43 is a characteristic sign of pathology in these diseases, it appears that it is the mislocalization of TDP-43 into the cytoplasm that confers toxicity, with dysfunctional RNA metabolism likely critical to pathogenesis. 

TDP-43 regulates the metabolism, splicing and transport of 1000s of mRNAs [90]. Expression of mutant TDP-43 decreased the motility of mRNP granules, reducing the transport of mRNAs, led to exon exclusion of some pre-mRNAs and increased normal splicing or translation of other targets [21,95,106,109,110]. In animal models expressing mutant TDP-43 deregulated splicing and alternative splicing was observed [109,110]. Thus, in ALS and FTLD cases where a *TARDBP* mutation leads to cytoplasmic mislocalization, or in sporadic cases where an unknown trigger causes translocation, the normal RNA regulatory functions of TDP-43 may be severely reduced and contribute to pathogenicity. Therefore, strategies to reduce TDP-43 nuclear depletion are an attractive approach to the effective treatment of ALS and FTLD. As deficiency and TDP-43 overexpression have profound effects on neuron viability and function [25,26,29,34], direct targeting of TDP-43 is a major roadblock to disease-modifying treatments. Therefore, targeting key factors that contribute to TDP-43 aggregation in neurons offers an alternative and original therapeutic approach. Further research to fully elucidate those triggers and identify the factors that trigger TDP-43 translocation are required. 

## 4. Conclusions

Overall, the literature suggests that TDP-43 proteinopathy occurs due to the translocation of TDP-43 out of the nucleus into the cytoplasm. This may occur due to stressors that induce ROS generation, or the presence of a gene mutation. Either may lead to a loss of nuclear function, including altered splicing and translation of mRNAs, and a gain of toxic functions in the cytoplasm, including dysregulated proteostasis, impaired mitochondria, activation of ER-induced cell death and impaired stress granule formation. The major unresolved questions are the following: are PTMs or TDP-43 misfolding a cause of disease or a result of pathogenesis? And do they induce TDP-43 translocation out of the nucleus? In answering these questions, we can tackle the problem of how to prevent PTMs and mislocalization from occurring. This will help to identify therapeutic targets, which may include chaperones involved in the folding of TDP-43.

## Figures and Tables

**Table 1:** Characteristics of mutant TDP-43 proteins and C-terminal fragments.

	Decreased Nuclear Localization	CTF Formed	Phosphorylation	Co-Aggregation with Wild Type	Increased Aggregation/Insolubility	Increased Half-Life
p.D169G	Y [73]			Y [71]	Y [42]	
p.K181E				Y [71]		
p.K263E				Y [71]		
p.G287S					N [42]	
p.G290A	N [28]	Y [28]	Y [28]		Y [28] N [42]	
p.S292N	Y [28]	Y [28]	Y [28]		Y [28]	
p.G294A					Y [42]	
p.G294V	N [28]	Y [28]	Y [28]		Y [28]	
p.G298S	N [28]	Y [28]	Y [28]		Y [28] N [42]	Y [28]
p.A315T	Y [28]	Y [28,50,74]	Y [28]		Y [28,48,51,74,75] N [42,74]	Y [28]
p.A315E	N [28]	Y [28]	Y [28]		Y [28]	
p.M331V	Y [28]	Y [28]	Y [28]		Y [28]	
p.S332N	N [28]	Y [28]	Y [28]		Y [28]	
p.M337V	Y [25] N [28]	Y [28]	Y [28]		Y [28,42,75] N [29,47]	Y [28]
p.Q331K	Y [25,55]			Y [55]	Y [42]	
p.Q343R	Y [28]	Y [28]	Y [28]		Y [28,75]	Y [28]
p.N345K	Y [28]	Y [28]	Y [28]		Y [28]	
p.G348C	N [28]	Y [28]	Y [28]		Y [28] N [42]	Y [28]
p.G348V	N [28]	Y [28]	Y [28]		Y [28]	
p.N352S	Y [28]	Y [28]	Y [28]		Y [28]	Y [28]
p.R361S					N [42]	
p.Y374X					Y [47]	
p.N378D	Y [28]	Y [28]	Y [28]		Y [28]	
p.S379P	Y [28]	Y [28]	Y [28]		Y [28]	
p.A382T	Y [28]	Y [28]	Y [28]		Y [28] N [29,42,48]	Y [28]
p.I383V	Y [28]	Y [28]	Y [28]		Y [28]	
p.N390D					Y [42]	
p.N390S					Y [42]	
p.S393L	Y [28]	Y [28]	Y [28]		Y [28]	
162-414		Y [45]				
167-414					N [47]	
173-414					N [47]	
178-414					N [47]	
183-414					N [47]	
188-414			Y [47]		single nuclear inclusion. Y [47]	
193-414			Y [47]		single nuclear inclusion. Y [47]	
198-414			Y [47]		single nuclear inclusion. Y [47]	
203-414			Y [47]		single nuclear inclusion. Y [47]	
208-414		Y [45]	Y [47,48,50]		multiple cytoplasmic inclusions. Y [47,48,50]	
213-414			Y [47]		multiple cytoplasmic inclusions. Y [47]	
220-414			Y [47]		multiple cytoplasmic inclusions. Y [47]	
225-414			Y [47]		multiple cytoplasmic inclusions. Y [47]	

## Data Availability

Not applicable.

## Abbreviations

aa	amino acid
ALS	amyotrophic lateral sclerosis
AMP	adenosine 3′, 5′-monophosphate
AMPA	α-amino-3-hydroxy-5-methyl-4-isoxazolepropinionic acid
AMPK	AMP-activated protein kinase
Ca^2+^	calcium
CK1	casein kinase 1ε
CTF	C-terminal fragment
eIF2α	eukaryotic initiation factor 2 alpha
EEAT2	excitatory amino acid transporter 2
ENU	* N * -ethyl-*N*-nitrosurea
ER	endoplasmic reticulum
FTD-TDP	frontotemporal degeneration associated with TDP-43 pathology
FTLD	frontotemporal lobar degeneration
G3BP	RasGAP-association endoribonuclease
G4-RNA	G-quadruplex-RNA
GSK-3β	glycogen synthase kinase 3β
LLPS	liquid–liquid phase separation
MALAT	metastasis-associated lung adenocarcinoma transcript 1 (also known as NEAT2; nuclear paraspeckle assembly transcript 2)
miRNA	micro RNA
mRNA	messenger ribonucleic acid
mTOR	mammalian target of rapamycin
NEAT1	nuclear paraspeckle assembly transcript 1
NES	nuclear export signal
NeuN	neuronal nuclei
neuroLNC	neuron-specific long-non-coding RNA
NLS	nuclear localization signal
NMJ	neuromuscular junction
PARP-1	poly-(ADP-ribose) polymerase-1
PINK1	PTEN-induced putative kinase 1
PP2A	protein phosphatase 2A
PSF	proline-glutamine rich
PTM	post-translational modification
RBP	RNA-binding protein
RNA	ribonucleic acid
RNS	reactive nitrogen species
ROS	reactive oxygen species
RRM	RNA recognition motif
SURF	SMG1, UPF1 and eRF1 and 2
TARDBP	TAR-DNA-binding protein 43
TBK1	tank-binding kinase 1
TDP-43	TAR-DNA-binding protein 43
TFEB	transcription factor EB
TIA-1	T-cell intracellular antigen 1
UPR	unfolded protein response
UPS	ubiquitin proteasome system
